# Framework for a new dialogue between psychoanalysis and neurosciences: is the combined neuro-psychoanalytic approach the missing link?

**DOI:** 10.1186/1747-5341-2-25

**Published:** 2007-11-01

**Authors:** Grigoris Vaslamatzis

**Affiliations:** 1 Department of Psychoanalytic Psychotherapy, Athens University Medical School, Eginition Hospital, V. Sophias Ave. 74, Athens, 11528, Greece

## Abstract

Freud's legacy deriving from his work The project for a scientific psychology (1895) could give a new impetus to the dialogue between psychoanalysis and neurosciences. A rapproachment phase is warrented. Based on the work of psychoanalysts who are themselves neuroscientists (such as Mauro Mancia, Martha Koukkou and Harold Shevrin) or have a long term dialogue with neuroscientists (Arnold Modell), three points of epistemological congruence are described:

1. dualism is no longer a satisfactory solution

2. cautions for the centrality of interpretation (hermeneutics)

3. the self-criticism of neuroscientists

## Introduction

"Psychoanalysis is unjustly reproached, gentlemen, for leading to purely psychological theories of pathological problems. The emphasis which it lays on the pathogenic role of sexuality, which, after all, is certainly not an exclusively psychical factor, should alone protect it from this reproach. Psychoanalysts never forget that the mental is based on the organic, although their work can only carry them as far as this basis and not beyond it." This is not my introduction. It is an excerpt from a lecture delivered by Freud in 1910[1]. At a considerable distance from his even earlier Project for a Scientific Psychology(1895) [2], reflection remains open. At the dusk of the nineteenth century, Freud had attempted to address the unsolved issue of the definition of mental function in terms of neural function or a wider connection of the psychic with brain function with his Project[2]. Having kept silent about this work himself, it was only published in 1950. Intriguingly, several neuroscientists and analysts interested in his ideas have recently attempted to revisit them in the context of contemporary knowledge. Mancia[3], reads more psychology rather than biology into it, a tendency to "mentalize" neurons. Others find different parts of the Freudian text interesting (for instance, as regards synapses and the time lag between entry and exit of sensory information, i.e. behaviour)[4,5] and believe they still carry weight today.

Of course, the issue with Freud's Project is not whether some of its positions have been confirmed whilst others are completely outdated. What matters is the legacy left behind for reflection and method. The integration of the findings of psychoanalysis with those of neuroscience remains an open question. Therefore, Freud's legacy can give a new impetus to the endeavour of reformulating, in the light of current knowledge, the assumption that "psychical processes run parallel to the physiological ones", that they are "dependent concomitant"[6]. It is the author's opinion that this legacy is understood by psychoanalysts who are themselves researchers in the field of neurosciences far better than by others. Shevrin[7], Mancia [3], and Koukkou[8] are inspired to integrate their two divergent disciplines: the discipline of the unconscious mind and the science of brain function. Alongside them is the recent work of Arnold Modell[9,10]. These authors speak in modern psychoanalytic language through the lens of the latest insights of neurobiology.

We are also aware that twenty years after the "Project" Freud would argue that "we are taken a step further – we do not know how much – by the discovery of the unequal importance of the different parts of the brain and their special relations to particular parts of the body and to particular mental activities. But every attempt to go on from there to discover a localization of mental processes, every endeavor to think of ideas as stored up in nervecells and of excitations are traveling along nerve-fibers has miscarried completely"[11]. This formulation reflects his fertile turn from the Project to the Interpretation of Dreams [12]. In taking this turn, he may be thought to have accepted again "dualism": body/psyche, brain/mind, nature/nurture. It was a new possibility that allowed:

a. the formulation of psychoanalytic methodology and theory,

b. the construction of the analytic object, and,

c. the development of a technique.

The basic links here are the unconscious and transference. One aspect of Freud's genius, as Spector-Person points out (1989) [13], is that he managed to remain a materialist although his only field of research was the unconscious. His biological suggestions had to wait for developments in the natural sciences. Freud held on firmly to this view as we know from his references in several texts. But he also oscillated constantly between dualism in etiology and an integrated model.

There are many analysts who argue that today psychoanalysis is once again approaching the neurosciences, but also the reverse, namely that it is neuroscientists who are looking for the lost "mind". An illustration of this can be found in Kandel's American Journal of Psychiatry article[14], which advances a framework for an encounter between psychoanalysis and neurobiology. Herein I propose a *rapprochement phase *between the two disciplines (I borrow the term from Mahler's work), following a long period of splitting, as occurs in developmental phases of the human being. Prompted by the writings of psychoanalyst/neuroscientists, three reasons for rapproachment spring to mind.

## About dualism in brain-mind dichotomy

First of all, in the field of epistemology, *dualism is no longer a satisfactory solution*. The fragmentation of human existence or psychoanalysis in complete isolation from the rest of thescientific world ("we study the psychological causes, the othersthe "organic" ones") can have no place in critical scientificdialogue, although endorsing dualism can be reassuring andinspiring for the treating psychoanalyst. In their routineanalytic work, psychoanalysts are concerned exclusively withmental function. However, the basis of so-called "mental" phenomena results from combined rather than independent processes in the brain and in the mind. Pointing out the complexity of coherence of brain and mind, Koukou posits experience dependent brain plasticity as a key concept for studying non-conscious decisions[8]. Molecular mechanisms "translate the human-specific experiences based on innate knowledge about what supports well-being and what disturbs well-being into the neural architecture of the experience-dependent synaptic plasticity (the cortico-cortical connectivity) of the neocortex and herewith extract personal meaning and create biography"[15]. This is how the plasticity of the brain, its ability to assimilate and dynamically interact with human experience, mainly autobiography, is understood. One might conclude that the idea that psychological problems are entirely the result of innate faults in the biological substratum follows from a failure of human sciences (and classical neuroscientists) to evaluatepsychoanalytic insights. Particularly those insights regarding theimportance of misunderstandings and dysfunctions in archaicrelationships of the individual with their environment for their dynamically developing brain.

The brain participates in the creation of human meaning. This insight has been developed by Modell as may be seen in his books *The Private Self*"[9] and *Imagination and the meaningful brain *[10]. The separation of science on the one hand from the interpretation of meaning on the other is flawed – not only because psychoanalysis brings the subject's meaning to the forefront of its investigation but also because by linking it to unconscious emotions it links it to a biological framework. Modell highlighted much earlier the importance of the Freudian theory of "nachträglichkeit", i.e. of the retranscription of memory in the psychoanalytic work of revealing meaning [9]. It is a theory that Edelman, a researcher of memory from the biological perspective, set out in the same terms for the central nervous system without having studied Freud. Memory is not a fixed transcription in the brain, isomorphic with past experience. Rather, it is a dynamic reconstruction associated with context and classified in categories [16]. Let us recall what Freud wrote to Fliess in 1896. "The material present in the shape of memory-traces is from time to time subjected to a rearrangement in accordance with fresh circumstances – is, as it were, transcribed. Thus, what is new in my theory is the thesis that memory is present not once but several times over, that it is registered in various species of "signs"[17]. Edelman's neural approach is, in this instance, congruent with Freud's psychoanalytic theory of memory and repetition. For Modell, these insights have implications for the theory of instincts and the mental apparatus as well, issues which are critical for psychoanalysts but which go beyond the scope of this paper [9].

Apart from humans, Modell cites modern research which demonstrates that meaning is fully individualized even in animals. [9] In humans, insights such as these imply emerging insights into the unconscious, and recall psychoanalyst Bollas formulation of the "unthought known" [18]: A mental state that has not become a thought yet but consists of non-verbal memories from archaic relationships which become known through the interaction of transference and counter transference and the analyst's "experiences". What are the implications of these insights? The "congruence" of psychoanalytic and neurobiological findings implies that neuroscientists should recognize what is psychoanalytically obvious (for instance, the "unthought unknown" or unconscious feelings) and can be meaningfully inspired by it or use it as a theoretical basis for integrated research.

This brings us, as Shevrin points out, to another epistemological problem: Neurobiological studies lack a "fundamental hypothesis," they lack integrity of "mind" and a "coherent story" of the subject[7]. Although human neurobiology belongs to the human sciences, often it does not talk about the human story. But equally, it is important for psychoanalysis to restore its relations with the biological and to do so in modern terms. Otherwise psychoanalysis runs the risk of being cut-off from the biological level and of becoming just another psychological theory. Psychoanalysis is at the same time a meta theory which by definition (by Freud himself) aims at connecting the biological substratum with mental functioning.

## Interpretation vs relationship

The second reason for rapproachment between psychoanalyis and neuroscience is the *limitation of the omnipotence of interpretation *(and, in this sense, the problem of a theological type of psychoanalysis). Motivation for rapproachment here comes from cultural change as well as from changes in methods of psychoanalytic research. Psychoanalysis itself is changing. There is now more recognition of:

a. resistance to change and the negative therapeutic reaction,

b. the contribution of preverbal traumas and of the earliest phases of development in psychopathology, and,

c. intersubjective construction of meaning.

Beyond a history of splits and differences in terminology which make the psychoanalytic community look like Babel, the above issues lie at the heart of this essay. This might be the reason that projective identification (both as a normal and a pathological process) has gradually touched almost every psychoanalyst. Mostly in the form of the "theoretical model" in which it was cast by Bion [19].

The analysand affects the analyst not only through associations but also through the feelings he/she projects. Projective identification is an alternative, albeit primitive, way of communication of feelings and impressions that a patient cannot tolerate and capture in words. Through this communication in the analytic situation we suppose that the patient will bring pressure to bear on the analyst. Bion described and distinguished normal projective identification as a primitive way of communication from different types of pathological (or excessive) projective identification [19,20]. The latter is at work when mind is evacuated of contents, as in the case of destructive and acting out behaviours or in hallucinations. Bion also assumed that there is a mental function of personality, calling it alpha-function, which transforms sense impressions and archaic feelings into elements of mental domain. These elements, called alpha elements, are the stuff of dream images in sleep or of unconscious thoughts in the state of wakefulness. What takes shape in the analyst's mind has to do also with the analysand's non-verbal "experience", especially when evacuation rather than thinking is at work. When alpha function is defective, as in the case of severe psychopathology, evacuation prevails (*via *pathological projective identifications) and internal processing of feelings is impossible.

Any change of the technique must be based on the analysts' ability to contain and process in their own minds and thus achieve a re-introjection by the patient of those mentally non-metabolized emotional and sensory stimuli. Something more than the interpretation of mental conflicts, this is the current consensus about what characterizes psychoanalysis as a therapy. More recently Gabbard and Westen emphasize that the therapeutic action of psychoanalysis depends more on *constructions *in the here and now of the analytic relationship than to reconstructions of the past [21].

With this "plasticity" of new hypotheses and individuation ("each patient is unique" or, as Modell put it, "we produce fantasies that are unique to us"), psychoanalysis remains and continues to be recognized by many as the most coherent and integrated model for investigating the "mind". In the Mancia's text it is argued that the task of the psychoanalyst today is to transform symbolically and render verbalizable the implicit mechanisms of the unrepressed unconscious. Verified in the neurobiological field, this thesis gives impetus to the clinical level, most of all, to the analytic approach to patients who do not change. The clinical example presented by Mancia confirms the possibility of achieving, in the course of analytic therapy, at a slow pace admittedly, acquisition of form and modification of those elements patients are unable to tell or remember.

## Neuroscientists searching for mind

The third reason for rapproachment, I presume, lies *in a certain self-criticism by neuroscientists*. I cannot argue with certainty that, following the "decade of the brain", supremacy of the model of reductionism ("mental phenomena are only conscious as products of neurons") and the feeling of omnipotence associated with it have retreated. However, at least judging from recent publications from Kandel and Shrevin, it would seem that their rivalry with Freud, typical of the last decade of the twentieth century, has begun to subside. Unconscious mental conflict exists, Shevrin tells us, for it has been demonstrated that on the biological level the so-called resting state of mind (lack of stimuli) is characterized, on the contrary, by intense neural activity and functioning. His experimental research is an invitation for cooperation between psychoanalysts and neuroscientists. Shevrin believes that this partnership, through integrated protocols, can advance knowledge of the "neuroscience of emotions"[7]. Finally I refer to Nagel who makes an integrated statement [22] "The mental and physiological concepts and their reference to this same inner phenomenon would then be seen as secondary and each partial in its grasp of the phenomenon: each would be seen as referring to something that extends beyond its ground of application".

Psychoanalysis and neurobiology speak different languages. Psychoanalysis studies the individual through an intersubjective relationship. This is its trade mark as a discipline and shall not be erased by a *rapprochement *with neurobiology. As Otto Kernberg said in his opening speech as president of the 39th Psychoanalytic Conference held in San Francisco (1995), there are currently two human sciences investigating the roots of human experience, psychoanalysis and neurobiology.

## A final comment

The investigations of authors working both as analysts and neuroscientists are going far beyond the level of general declarations. We are presented with well documented proposals on how to combine the insights gained from the study of the brain with psychoanalytic research. Each author cited here provides us with knowledge and direction from their outstanding interdisciplinary research. What is striking is that their investigations, independent from each other, meet frequently at several points. Freud's "Project" (and I mean it in the sense of "legacy") is no longer utopian.

## Competing interests

The author(s) declare that they have no competing interests.
